# Supplementary material for: Global patterns and predictors of C:N:P in marine ecosystems
Source: Commun Earth Environ. 2022 Nov 7;3(1):271. doi: 10.1038/s43247-022-00603-6 (PMC9640808; doi:10.1038/s43247-022-00603-6)
Supplement: Supplementary file 1 — Supplementary Information [file 43247_2022_603_MOESM1_ESM.pdf]

Supplementary Information for:

# Global patterns and predictors of C:N:P in marine ecosystems

Tatsuro Tanioka[1], Catherine A. Garcia[1,2], Alyse A. Larkin[1], Nathan S. Garcia[1], Adam J. Fagan[1], and Adam C. Martiny[1,3,*]

[1] Department of Earth System Science, University of California Irvine, Irvine, California 92697, USA
[2] Center for Microbial Oceanography: Research and Education (C-MORE), University of Hawaii at Manoa, Honolulu, Hawaii 96822, USA
[3] Department of Ecology and Evolutionary Biology, University of California Irvine, Irvine, California 92697, USA
[*] Corresponding author (amartiny@uci.edu)

## Supplementary Figures

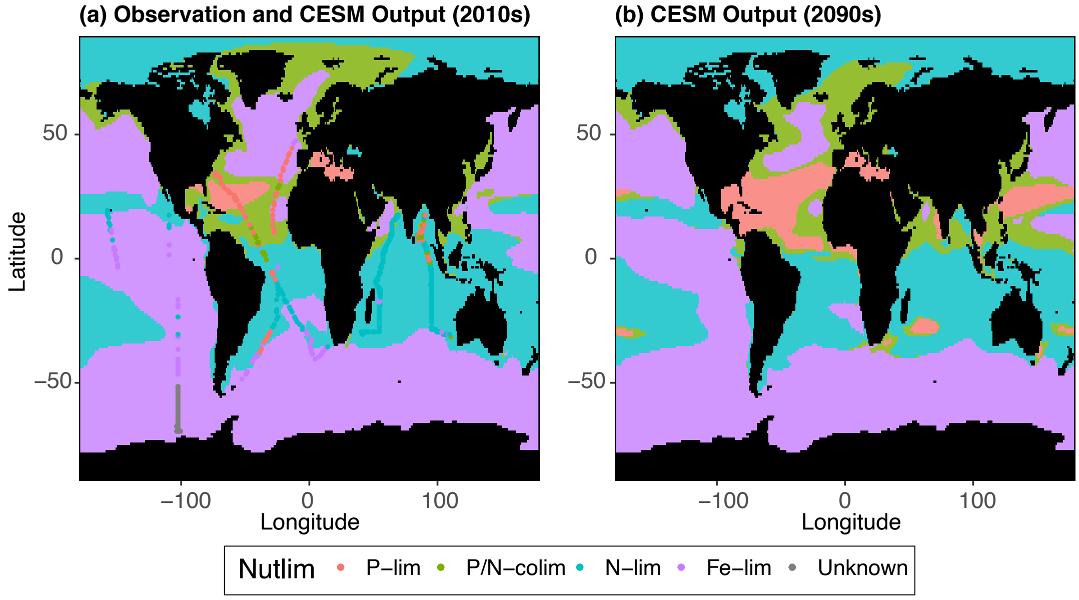


**Supplementary Figure 1: Global map of observed and modeled nutrient limitation type for small phytoplankton.** Different colors indicate different nutrient limitation types, and the dots are metagenome-derived nutrient limitation types for corresponding stations in which POM samples were collected (Purple = Fe-limited, Blue = N-limited, Green = P/N co-limited, Red = P-limited, Grey = Not Applicable). Shadings are based on nutrient limitation of small phytoplankton types from the Community Earth System Model Large Ensemble Simulation (CESM2-LENS) for (**a**) the 2010s and (**b**) the 2090s under SSP3-7.0 scenario.

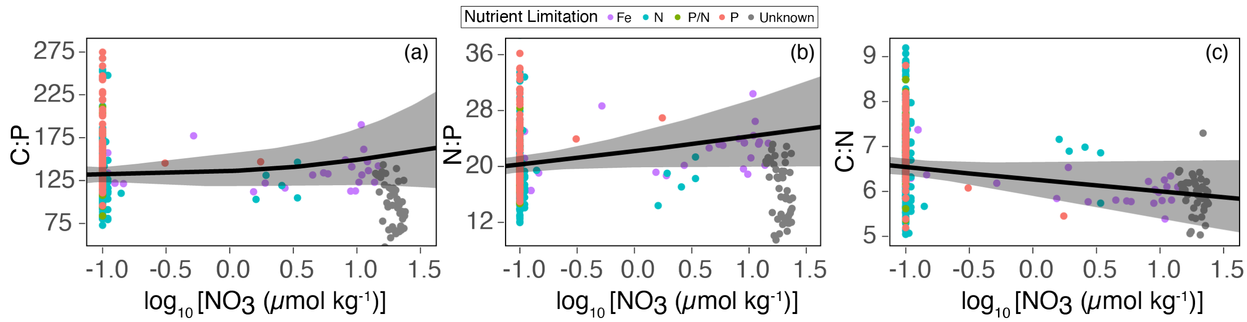


**Supplementary Figure 2: Observed C:N:P as a function of surface nitrate concentration for (a) C:P, (b) N:P, and (c) C:N.** For GAM predictions shown in black lines and shades, SST and nutricline were kept constant at the observed mean values of 25 °C and 70 m, respectively. Dots are observed values, and colors represent the nutrient limitation type inferred from metagenomes (Purple = Fe-limited, Blue = N-limited, Green = P/N co-limited, Red = P-limited, Grey = Unknown).

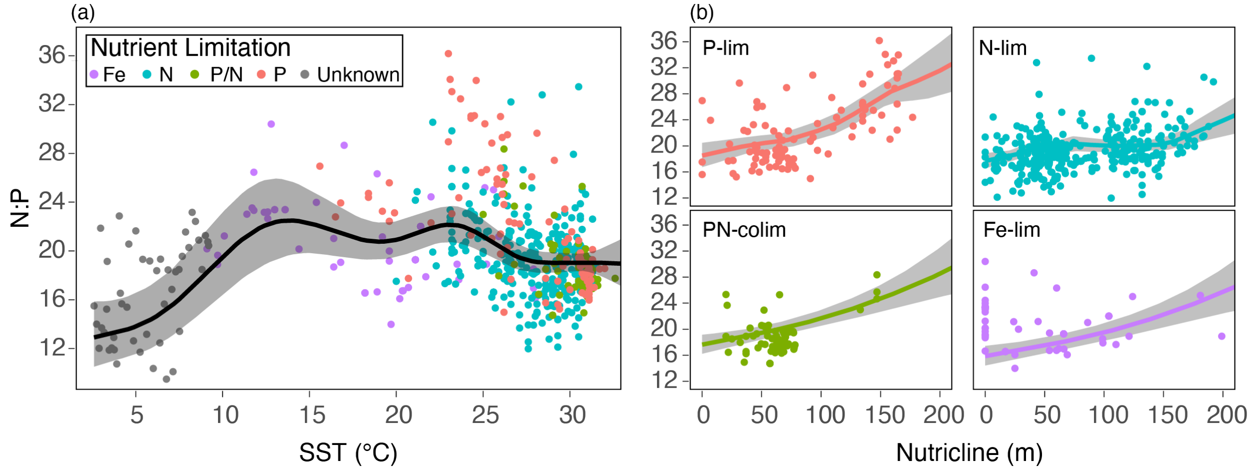


**Supplementary Figure 3: Observed N:P as a function of environmental variation**. Dots are observed values, and colors represent the nutrient limitation type inferred from metagenomes (Purple = Fe-limited, Blue = N-limited, Green = P/N co-limited, Red = P-limited, Grey = Unknown). (**a**) N:P against SST. The black line and shade represent GAM prediction and uncertainty (± 2SE) under the constant nutricline depth and surface nitrate values at the observed mean values of 70 m and 0.2 μmol $kg^{-1}$, respectively. (**b**) N:P against nutricline depth for different nutrient limitation types. GAM is fitted separately for each limiting nutrient type under constant SST and surface nitrate at the observed mean values of 25 °C and 0.2 μmol $kg^{-1}$, respectively.

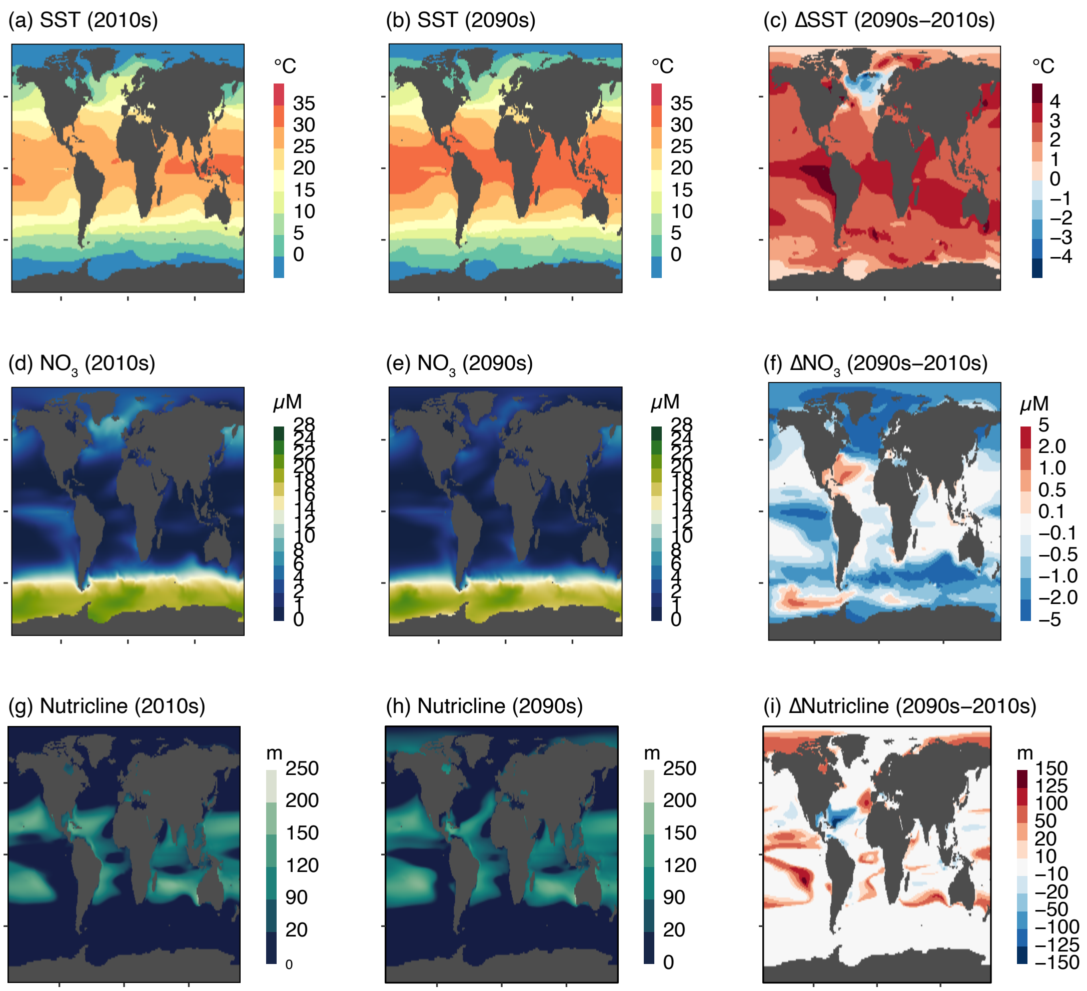


**Supplementary Figure 4: Modeled annual mean nutrient, temperature, and nutricline fields from CESM2-LENS for the historic periods (2010s) and the 2090s under the SSP3-7.0 scenario.** The last column shows the difference between the 2090s and 2010s.

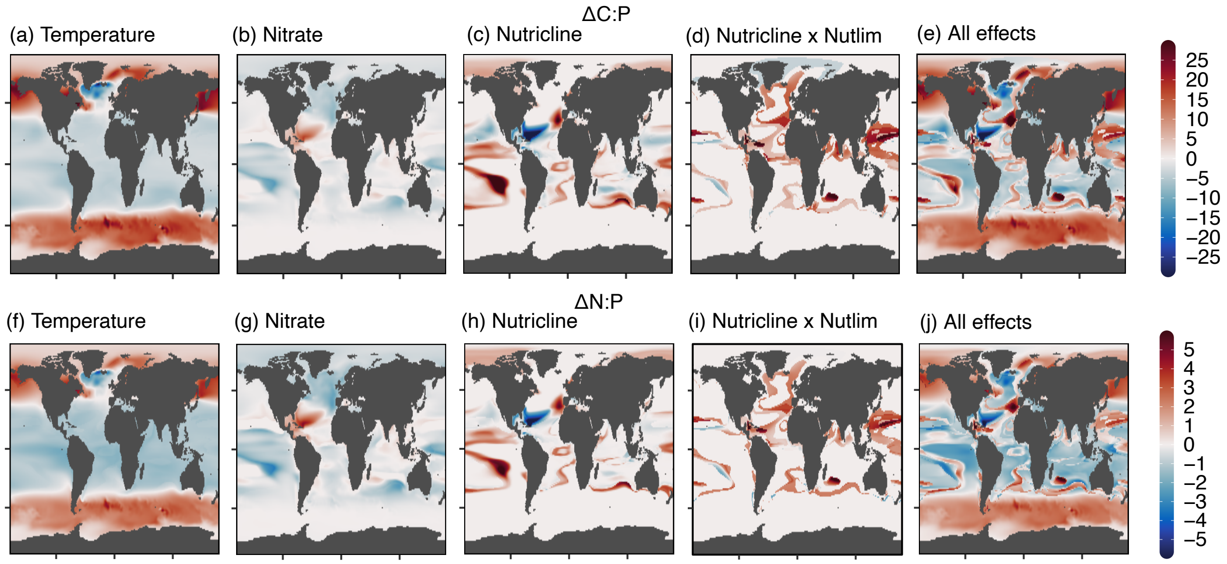


**Supplementary Figure 5: Evaluation of drivers of future C:P and N:P change.** Impact of changes in (**a, f**) SST, (**b, g**) surface nitrate concentration, (**c, h**) nutricline depth, (**d, i**) interaction between nutricline and nutrient limitation, and (**e, j**) the combined effects for the change in community C:P and N:P from the 2010s to the 2090s under the SSP3-7.0 scenario.

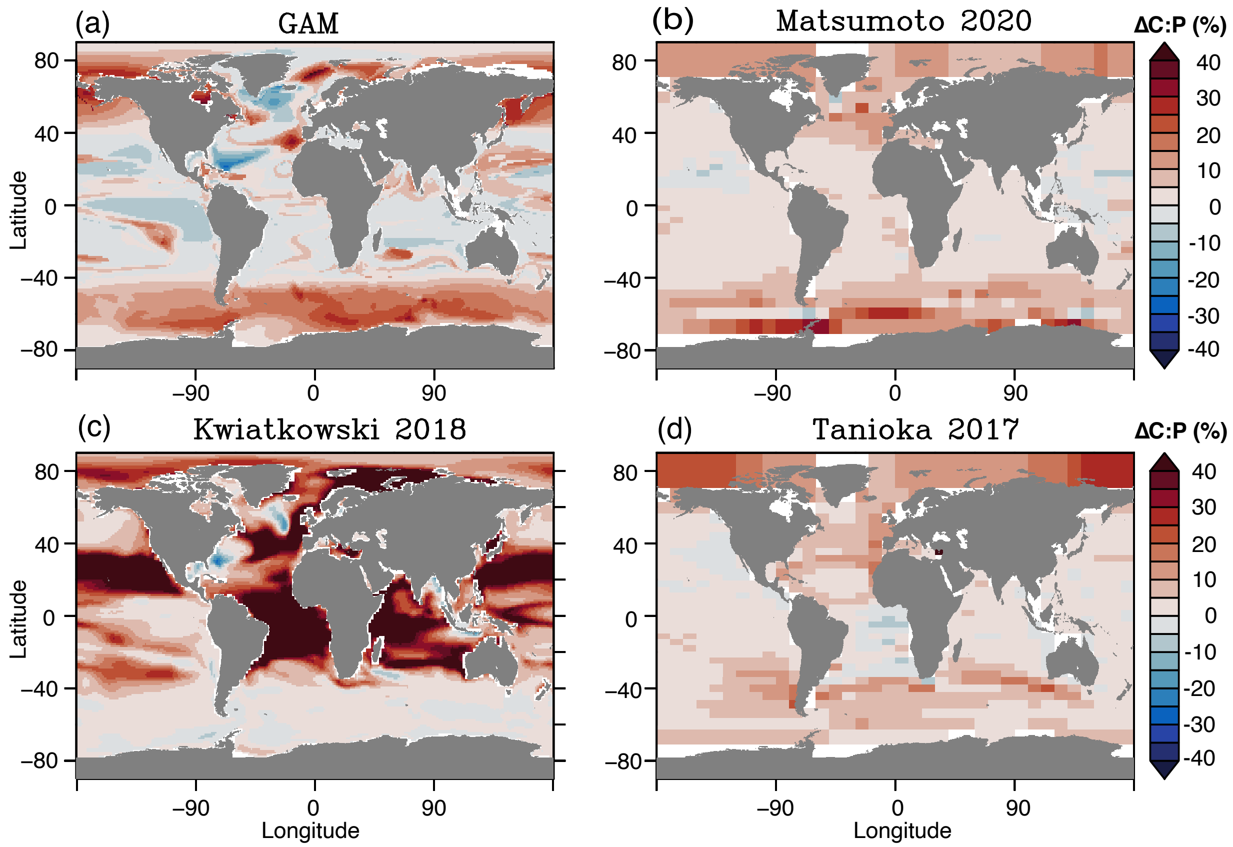


**Supplementary Figure 6: Projections of change in C:P under future conditions for different models.** (**a**) % change in C:P projected with data-driven GAM applied to output from CESM2 Large Ensemble simulation under SSP3-7.0 scenario from the 2010s to 2090s, (**b**) % change in model C:P export ratio of POM at 100 m under SSP2 scenario from 2000 to 2100 with MESMO3, (**c**) % change in model suspended C:P ratio of POM at 100 m under RCP8.5 scenario from 2010s to 2090s with PISCES-QUOTA, (**d**) % change in model C:P export at 100 m under RCP8.5 scenario from 2000 to 2090s with MESMO2.

## Supplementary Tables

**Supplementary Table 1: Surface POM samples from Bio-GO-SHIP cruises used in this study**. All POM data used in this study are publicly available. *C13.5 is a partial occupation of the A13.5 GO-SHIP line that was aborted due to COVID-19. See Larkin et al. (2021)[28] for further details on metadata variables.

| Campaign | Temporal Coverage | Spatial Coverage | DOI | n |
|---|---|---|---|---|
| AMT-28, PML AMT SOCCOM, NSF | 25 September 2018 - 27 October 2018 | Meridional Atlantic Transect (N:49.6380 E:-5.5020 S: -48.1990 W:-52.692) | https://doi.org/10/fmkq | 740 |
| C13.5*, GO-SHIP | 21 March 2020 – 16 April 2020 | Meridional Atlantic Transect (N: 34.5044 E:17.3055 S:-41.4917 W:-73.51) | https://doi.org/10.26008/1912/bco-dmo.868908.1 | 118 |
| I07N, GO-SHIP | 25 April 2018 – 04 June 2018 | Western Indian Ocean (N:17.9990 E:69.4830 S:-30.0120 W:40.0080) | https://doi.org/10.26008/1912/bco-dmo.879076.1 | 661 |
| I09N, GO-SHIP | 22 March 2016 – 24 April 2016 | Eastern Indian Ocean (N:17.8831 E:110.4547 S:-31.0335 W:84.7526) | https://doi.org/10.26008/1912/bco-dmo.734915.3 | 235 |
| NH1418, NSF | 20 September 2014 – 6 October 2014 | Tropical Pacific Ocean (N:19.0010 E:-149.6700 S:-3.0001 W:-158.0000) | https://doi.org/10.26008/1912/bco-dmo.829895.1 | 21 |
| P18, GO-SHIP | 13 November 2016 – 28 January 2017 | Meridional Pacific Transect (N:28.9071 E:-100.3721 S:-69.9027 W:-116.0561) | https://doi.org/10.26008/1912/bco-dmo.816347.1 | 195 |

**Supplementary Table 2: Area-weighted global mean C:N:P (Observation)**

| | **Mean** | **l-95% CI** | **u-95% CI** | **n** |
|---|---|---|---|---|
| C:P | 137.3 | 135.9 | 138.8 | 1970 |
| N:P | 20.7 | 20.6 | 20.9 | 1970 |
| C:N | 6.62 | 6.58 | 6.65 | 1970 |

**Supplementary Table 3: Area-weighted global mean C:N:P (Observation, binned by 1-by-1 grid)**

| | **Mean** | **l-95% CI** | **u-95% CI** | **n** |
|---|---|---|---|---|
| C:P | 142.3 | 139.8 | 144.8 | 509 |
| N:P | 21.2 | 20.9 | 21.5 | 509 |
| C:N | 6.72 | 6.65 | 6.78 | 509 |

**Supplementary Table 4: Regionally-binned, area-weighted C:N:P.** Polar (|Latitude| ≥ 65°), Subpolar (45° ≤ |Latitude| < 65°), Subtropical (15° ≤ |Latitude| < 45°), and Tropical (|Latitude| < 15°). Mean and 95% confidence intervals were computed for log-transformed C:N:P ratios and were converted back by taking the exponential.

| **Region** | **Mean** | **Median** | **l-95% CI** | **u-95% CI** | **n** |
|---|---|---|---|---|---|
| **C:P** | | | | | |
| Polar | 82.8 | 77.7 | 63.1 | 125.3 | 16 |
| Subpolar | 133.7 | 134.7 | 65.5 | 198.0 | 129 |
| Subtropical | 148.1 | 147.5 | 64.5 | 228.6 | 1005 |
| Tropical | 129.4 | 126 | 70.5 | 185 | 820 |
| **N:P** | | | | | |
| Polar | 14.1 | 13.2 | 10.1 | 19.1 | 16 |
| Subpolar | 21.5 | 21.6 | 15.2 | 28.7 | 129 |
| Subtropical | 22.1 | 22.0 | 12.5 | 31.1 | 1005 |
| Tropical | 19.7 | 19.0 | 11.8 | 27.5 | 820 |
| **C:N** | | | | | |
| Polar | 5.7 | 5.7 | 5.4 | 6.1 | 16 |
| Subpolar | 6.2 | 6.1 | 5.0 | 7.3 | 129 |
| Subtropical | 6.7 | 6.7 | 5.0 | 8.9 | 1005 |
| Tropical | 6.6 | 6.6 | 5.0 | 8.4 | 820 |

**Supplementary Table 5: Correlation matrix for (Sub)polar region C:N:P**. Asterisks represent the statistical significance (***: $p < 0.001$, **: $p < 0.01$, *: $p < 0.05$, NA: Not Applicable).

| | **Absolute Latitude** | **SST** | **Nitrate** | **Phosphate** | **FeT** | **Nutricline** | **MLD** | **MLPAR** | **Chl-a** | **% Diatoms** | **% Coccolithophores** | **% Chlorophytes** | **% Cyanobacteria** |
|---|---|---|---|---|---|---|---|---|---|---|---|---|---|
| C:P | -0.79 (***) | 0.80 (***) | -0.71 (***) | -0.72 (***) | 0.65 (***) | 0.62 (***) | -0.11 | -0.31 (*) | 0.65 (***) | -0.76 (***) | 0.77 (***) | -0.08 | 0.68 (***) |
| N:P | -0.65 (***) | 0.66 (***) | -0.57 (***) | -0.58 (***) | 0.54 (***) | 0.48 (**) | -0.19 | -0.22 | 0.53 (***) | -0.63 (***) | 0.63 (***) | -0.09 | 0.56 (***) |
| C:N | -0.55 (***) | 0.58 (***) | -0.55 (***) | -0.54 (***) | 0.45 (**) | 0.52 (***) | 0.17 | -0.31 (*) | 0.49 (**) | -0.55 (***) | 0.55 (***) | 0.01 | 0.51 (***) |

**Supplementary Table 6: Correlation matrix for (Sub)tropical region C:N:P**.

| | **Absolute Latitude** | **SST** | **Nitrate** | **Phosphate** | **FeT** | **Nutricline** | **MLD** | **MLPAR** | **Chl-a** | **% Diatoms** | **% Coccolithophores** | **% Chlorophytes** | **% Cyanobacteria** |
|---|---|---|---|---|---|---|---|---|---|---|---|---|---|
| C:P | 0.23 (***) | -0.11 (***) | -0.19 (***) | -0.29 (***) | -0.03 | 0.45 (***) | -0.12 (***) | 0.22 (***) | -0.28 (***) | -0.12 (***) | 0.01 | -0.31 (***) | 0.35 (***) |
| N:P | 0.29 (***) | -0.24 (***) | -0.02 | -0.15 (***) | -0.08 (**) | 0.29 (***) | -0.01 | 0.05 | -0.11 (***) | -0.03 | 0.11 (***) | -0.32 (***) | 0.17 (***) |
| C:N | -0.04 | 0.20 (***) | -0.36 (***) | -0.32 (***) | 0.09 (**) | 0.39 (***) | -0.23 (***) | 0.36 (***) | -0.38 (***) | -0.28 (***) | -0.17 (***) | -0.07 (*) | 0.41 (***) |

**Supplementary Table 7: The individual explained deviance and additive contribution of the four main contextual variables to the total explained deviance in generalized additive models (GAMs) for (Sub)polar region C:N:P.** Asterisks represent the statistical significance (***: $p < 0.001$, **: $p < 0.01$, *: $p < 0.05$).

| | **SST** | **Nitrate** | **Nutricline** | **Nutricline x Nutlim** | **Total Explained Deviance** |
|---|---|---|---|---|---|
| C:P | 0.367 (***) | 0.115 | 0.065 | 0 | 0.548 |
| N:P | 0.303 (***) | 0.103 | 0.056 | 0 | 0.463 |
| C:N | 0.060 (**) | 0.031 (*) | 0.028 (*) | 0 | 0.118 |

**Supplementary Table 8: As in Table S7 but for (Sub)tropical region C:N:P.**

| | **SST** | **Nitrate** | **Nutricline** | **Nutricline x Nutlim** | **Total Explained Deviance** |
|---|---|---|---|---|---|
| C:P | 0.042 (*) | 0.010 | 0.201 (***) | 0.139 (**) | 0.392 |
| N:P | 0.072 (***) | 0.019 (**) | 0.100 (***) | 0.178 (***) | 0.370 |
| C:N | 0.022 (*) | 0.014 | 0.027 | 0.073 (***) | 0.136 |

**Supplementary Table 9: Regionally-binned, area-weighted mean C:P and N:P under the SSP3-7.0 and historic CMIP6 scenarios.**

| **Region** | **C:P Historical (2010s)** | **C:P SSP3-7.0 (2090s)** | **N:P Historical (2010s)** | **N:P SSP3-7.0 (2090s)** |
|---|---|---|---|---|
| Polar (\|Latitude\| ≥ 65°) | 59.6 | 65.5 | 10.4 | 11.3 |
| Subpolar (45° ≤ \|Latitude\| < 65°) | 92.2 | 104.7 | 15.6 | 17.5 |
| Subtropical (15° ≤ \|Latitude\| < 45°) | 141.0 | 141.9 | 22.1 | 21.9 |
| Tropical (\|Latitude\| < 15°) | 129.9 | 129.4 | 20.0 | 19.0 |
| (Sub)polar (\|Latitude\| ≥ 45°) | 83.2 | 94.2 | 14.2 | 15.9 |
| (Sub)tropical (\|Latitude\| < 45°) | 136.7 | 137.0 | 21.3 | 20.7 |
| Global | 120.1 | 124.4 | 19.2 | 19.4 |

**Supplementary Table 10: Model fits for different hierarchical GAM models for C:P, ordered based on AIC and RMSE.**
**(*df* = degrees of freedom, AIC = Akaike Information criterion, ΔAIC = difference in AIC from the model with lowest AIC, RMSE = Root mean squared error, $RMSE_{norm}$ = RMSE normalized by mean value, $R^2$ (train) = coefficient of determination for training data, $R^2$ (test) = coefficient of determination for validation data).**

| **Model** | ***df*** | **AIC** | **ΔAIC** | **RMSE** | **$RMSE_{norm}$** | **$R^2$ (train)** | **$R^2$ (test)** |
|---|---|---|---|---|---|---|---|
| GI | 17 | -406 | 0 | 0.169 | 0.034 | 0.392 | 0.324 |
| I | 19 | -403 | 3 | 0.172 | 0.035 | 0.391 | 0.297 |
| S | 19 | -403 | 3 | 0.170 | 0.035 | 0.389 | 0.313 |
| GS | 16 | -404 | 2 | 0.169 | 0.034 | 0.387 | 0.324 |
| G | 12 | -399 | 7 | 0.168 | 0.034 | 0.367 | 0.336 |
| C | 9 | -355 | 51 | 0.174 | 0.036 | 0.308 | 0.282 |

**Supplementary Table 11: As in Table 10 for N:P**

| **Model** | ***df*** | **AIC** | **ΔAIC** | **RMSE** | **$RMSE_{norm}$** | **$R^2$ (train)** | **$R^2$ (test)** |
|---|---|---|---|---|---|---|---|
| GI | 20 | -420 | 3 | 0.167 | 0.056 | 0.368 | 0.282 |
| I | 17 | -423 | 0 | 0.169 | 0.057 | 0.358 | 0.263 |
| GS | 18 | -420 | 3 | 0.168 | 0.056 | 0.358 | 0.276 |
| S | 18 | -420 | 3 | 0.167 | 0.056 | 0.358 | 0.276 |
| G | 11 | -385 | 38 | 0.169 | 0.057 | 0.300 | 0.261 |
| C | 8 | -346 | 77 | 0.175 | 0.059 | 0.240 | 0.214 |

**Supplementary Table 12: As in Table 10 for C:N**

| **Model** | ***df*** | **AIC** | **ΔAIC** | **RMSE** | **$RMSE_{norm}$** | **$R^2$ (train)** | **$R^2$ (test)** |
|---|---|---|---|---|---|---|---|
| GI | 18 | -785 | 0 | 0.119 | 0.062 | 0.145 | 0.103 |
| I | 15 | -783 | 2 | 0.119 | 0.062 | 0.134 | 0.093 |
| GS | 13 | -784 | 1 | 0.119 | 0.062 | 0.133 | 0.100 |
| S | 13 | -783 | 2 | 0.119 | 0.062 | 0.130 | 0.098 |
| G | 11 | -775 | 11 | 0.120 | 0.063 | 0.107 | 0.088 |
| C | 8 | -772 | 13 | 0.120 | 0.063 | 0.097 | 0.084 |

## Supplementary Methods

### Additional Information on Hierarchical GAM analysis

As the nutrient limitation information is a categorical variable, we first conducted hierarchical GAM analysis using the R package *mgcv*[61] to determine whether the shape of the function itself varies with different nutrient limitations. Based on the initial data exploration, we hypothesized that the response of CNP with respect to the change in nutricline depth varies with respect to the nutrient limitation type and compared with the null model that the responses are the same for all the nutrient limitation types. Following the standard methodology[62], we computed root-mean-squared-error and AIC of 6 interactive GAM models (including the control) by comparing model fit with the observation:

1. Model G = A global smoother (shape) for all observations
2. Model GS = Single common smoother plus group-level smoothers that have the same wiggliness
3. Model GI = Single common smoother plus group-level smoothers that have the different wiggliness
4. Model S = Group-specific smoothers (shape) without a global smoother, but all smoothers have the same wiggliness.
5. Model I = Group-specific smoothers with different wiggliness
6. Model C = Control, no dependence on nutrient limitation types

To compare the performance of each model, we conducted cross-validation (100 random partitions holding out 20% of observations) and found that model GI performed best over other models in terms of AIC, root-mean-squared error, and the coefficient of determination (Supplementary Tables S10-12). Thus, throughout this paper, we decided to use the model GI to describe the interaction between nutricline and element-specific nutrient limitations.

**Supplementary References**


1. Redfield, A. C., Ketchum, B. H. & Richards, F. A. The influence of organisms on the composition of Seawater. in *The composition of seawater: Comparative and descriptive oceanography. The sea: ideas and observations on progress in the study of the seas* (ed. Hill, M. N.) vol. 2 26–77 (Interscience Publishers, 1963).
2. Deutsch, C. & Weber, T. Nutrient Ratios as a Tracer and Driver of Ocean Biogeochemistry. *Ann. Rev. Mar. Sci.* **4**, 113–141 (2012).
3. Martiny, A. C. *et al.* Strong latitudinal patterns in the elemental ratios of marine plankton and organic matter. *Nat. Geosci.* **6**, 279–283 (2013).
4. Teng, Y.-C., Primeau, F. W., Moore, J. K., Lomas, M. W. & Martiny, A. C. Global-scale variations of the ratios of carbon to phosphorus in exported marine organic matter. *Nat. Geosci.* **7**, 895–898 (2014).
5. Garcia, C. A. *et al.* Nutrient supply controls particulate elemental concentrations and ratios in the low latitude eastern Indian Ocean. *Nat. Commun.* **9**, 4868 (2018).
6. Garcia, N. S. *et al.* The diel cycle of surface ocean elemental stoichiometry has implications for ocean productivity. *Global Biogeochem. Cycles* e2021GB007092 (2022) doi:10.1029/2021GB007092.
7. Singh, A. *et al.* C : N : P stoichiometry at the Bermuda Atlantic Time-series Study station in the North Atlantic Ocean. *Biogeosciences* **12**, 6389–6403 (2015).
8. Karl, D. M. *et al.* Ecological nitrogen-to-phosphorus stoichiometry at station ALOHA. *Deep Sea Res. Part II Top. Stud. Oceanogr.* **48**, 1529–1566 (2001).
9. Fagan, A. J., Moreno, A. R. & Martiny, A. C. Role of ENSO Conditions on Particulate Organic Matter Concentrations and Elemental Ratios in the Southern California Bight. *Front. Mar. Sci.* **6**, 386 (2019).
10. Matsumoto, K., Tanioka, T. & Rickaby, R. Linkages Between Dynamic Phytoplankton C:N:P and the Ocean Carbon Cycle Under Climate Change. *Oceanography* **33**, 44–52 (2020).
11. Galbraith, E. D. & Martiny, A. C. A simple nutrient-dependence mechanism for predicting the stoichiometry of marine ecosystems. *Proc. Natl. Acad. Sci.* **112**, 8199–8204 (2015).
12. Wang, W.-L., Moore, J. K., Martiny, A. C. & Primeau, F. W. Convergent estimates of marine nitrogen fixation. *Nature* **566**, 205–211 (2019).
13. Mills, M. M. & Arrigo, K. R. Magnitude of oceanic nitrogen fixation influenced by the nutrient uptake ratio of phytoplankton. *Nat. Geosci.* **3**, 412–416 (2010).
14. Kwiatkowski, L., Aumont, O., Bopp, L. & Ciais, P. The Impact of Variable Phytoplankton Stoichiometry on Projections of Primary Production, Food Quality, and Carbon Uptake in the Global Ocean. *Global Biogeochem. Cycles* **32**, 516–528 (2018).
15. Sterner, R. W. & Elser, J. J. *Ecological stoichiometry: the biology of elements from molecules to the biosphere*. (Princeton University Press, 2002).
16. Allen, A. P. & Gillooly, J. F. Towards an integration of ecological stoichiometry and the metabolic theory of ecology to better understand nutrient cycling. *Ecol. Lett.* **12**, 369–384 (2009).
17. Moreno, A. R. & Martiny, A. C. Ecological Stoichiometry of Ocean Plankton. *Ann. Rev. Mar. Sci.* **10**, 43–69 (2018).
18. Schaum, C.-E., Buckling, A., Smirnoff, N., Studholme, D. J. & Yvon-Durocher, G. Environmental fluctuations accelerate molecular evolution of thermal tolerance in a marine diatom. *Nat. Commun.* **9**, 1719 (2018).

19. Geider, R. & La Roche, J. Redfield revisited: variability of C:N:P in marine microalgae and its biochemical basis. *Eur. J. Phycol.* **37**, 1–17 (2002).

20. Sharoni, S. & Halevy, I. Nutrient ratios in marine particulate organic matter are predicted by the population structure of well-adapted phytoplankton. *Sci. Adv.* **6**, eaaw9371 (2020).

21. Lomas, M. W. *et al.* Varying influence of phytoplankton biodiversity and stoichiometric plasticity on bulk particulate stoichiometry across ocean basins. *Commun. Earth Environ.* **2**, 143 (2021).

22. Quigg, A. *et al.* The evolutionary inheritance of elemental stoichiometry in marine phytoplankton. *Nature* **425**, 291–4 (2003).

23. Yvon-Durocher, G., Dossena, M., Trimmer, M., Woodward, G. & Allen, A. P. Temperature and the biogeography of algal stoichiometry. *Glob. Ecol. Biogeogr.* **24**, 562–570 (2015).

24. Martiny, A. C. *et al.* Biogeochemical controls of surface ocean phosphate. *Sci. Adv.* **5**, eaax0341 (2019).

25. Moore, C. M. *et al.* Processes and patterns of oceanic nutrient limitation. *Nat. Geosci.* **6**, 701–710 (2013).

26. Klausmeier, C. A., Litchman, E. & Levin, S. A. Phytoplankton growth and stoichiometry under multiple nutrient limitation. *Limnol. Oceanogr.* **49**, 1463–1470 (2004).

27. Garcia, N. S., Bonachela, J. A. & Martiny, A. C. Interactions between growth-dependent changes in cell size, nutrient supply and cellular elemental stoichiometry of marine Synechococcus. *ISME J.* **10**, 1–10 (2016).

28. Larkin, A. A. *et al.* High spatial resolution global ocean metagenomes from Bio-GO-SHIP repeat hydrography transects. *Sci. Data* **8**, 107 (2021).

29. Clayton, S. *et al.* Bio-GO-SHIP: The Time Is Right to Establish Global Repeat Sections of Ocean Biology. *Front. Mar. Sci.* **8**, (2022).

30. Martiny, A. C., Vrugt, J. A., Primeau, F. W. & Lomas, M. W. Regional variation in the particulate organic carbon to nitrogen ratio in the surface ocean. *Global Biogeochem. Cycles* **27**, 723–731 (2013).

31. Ustick, L. J. *et al.* Metagenomic analysis reveals global-scale patterns of ocean nutrient limitation. *Science (80-. ).* **372**, 287–291 (2021).

32. Rodgers, K. *et al.* Ubiquity of human-induced changes in climate variability. *Earth Syst. Dyn. Discuss.* 1–22 (2021) doi:10.5194/ESD-2021-50.

33. O'Neill, B. C. *et al.* A new scenario framework for climate change research: the concept of shared socioeconomic pathways. *Clim. Change* **122**, 387–400 (2014).

34. Matsumoto, K. & Tanioka, T. Shifts in regional production as a driver of future global ocean production stoichiometry. *Environ. Res. Lett.* **15**, 124027 (2020).

35. Tanioka, T. & Matsumoto, K. Buffering of Ocean Export Production by Flexible Elemental Stoichiometry of Particulate Organic Matter. *Global Biogeochem. Cycles* **31**, 1528–1542 (2017).

36. Flombaum, P., Wang, W.-L., Primeau, F. W. & Martiny, A. C. Global picophytoplankton niche partitioning predicts overall positive response to ocean warming. *Nat. Geosci.* **13**, 116–120 (2020).

37. Cermeno, P. *et al.* The role of nutricline depth in regulating the ocean carbon cycle. *Proc. Natl. Acad. Sci.* **105**, 20344–20349 (2008).

38. Gregg, W. W. & Casey, N. W. Sampling biases in MODIS and SeaWiFS ocean chlorophyll data. *Remote Sens. Environ.* **111**, 25–35 (2007).

39. Gregg, W. W., Ginoux, P., Schopf, P. S. & Casey, N. W. Phytoplankton and iron: validation of a global three-dimensional ocean biogeochemical model. *Deep Sea Res. Part II Top. Stud. Oceanogr.* **50**, 3143–3169 (2003).
40. Toseland, A. *et al.* The impact of temperature on marine phytoplankton resource allocation and metabolism. *Nat. Clim. Chang.* **3**, 979–984 (2013).
41. Sauterey, B. & Ward, B. A. Environmental control of marine phytoplankton stoichiometry in the North Atlantic Ocean. *Proc. Natl. Acad. Sci.* **119**, e2114602118 (2022).
42. Marañón, E., Lorenzo, M. P., Cermeño, P. & Mouriño-Carballido, B. Nutrient limitation suppresses the temperature dependence of phytoplankton metabolic rates. *ISME J.* **12**, 1836–1845 (2018).
43. Thomas, M. K. *et al.* Temperature–nutrient interactions exacerbate sensitivity to warming in phytoplankton. *Glob. Chang. Biol.* **23**, 3269–3280 (2017).
44. Droop, M. R. The nutrient status of algal cells in continuous culture. *J. Mar. Biol. Assoc. United Kingdom* **54**, 825–855 (1974).
45. Hillebrand, H. *et al.* Goldman revisited: Faster growing phytoplankton has lower N:P and lower stoichiometric flexibility. *Limnol. Oceanogr.* **58**, 2076–2088 (2013).
46. Gruber, N. & Sarmiento, J. L. Global patterns of marine nitrogen fixation and denitrification. *Global Biogeochem. Cycles* **11**, 235–266 (1997).
47. Moore, J. K., Doney, S. C. & Lindsay, K. Upper ocean ecosystem dynamics and iron cycling in a global three-dimensional model. *Global Biogeochem. Cycles* **18**, (2004).
48. Weber, T. S. & Deutsch, C. Oceanic nitrogen reservoir regulated by plankton diversity and ocean circulation. *Nature* **489**, 419–422 (2012).
49. Barton, S. *et al.* Evolutionary temperature compensation of carbon fixation in marine phytoplankton. *Ecol. Lett.* **23**, 722–733 (2020).
50. Liang, Z., Letscher, R. T. & Knapp, A. N. Dissolved organic phosphorus concentrations in the surface ocean controlled by both phosphate and iron stress. *Nat. Geosci.* 1–7 (2022) doi:10.1038/s41561-022-00988-1.
51. Moran, M. A. *et al.* Deciphering ocean carbon in a changing world. *Proc. Natl. Acad. Sci.* **113**, 3143–3151 (2016).
52. Flombaum, P. *et al.* Present and future global distributions of the marine Cyanobacteria Prochlorococcus and Synechococcus. *Proc. Natl. Acad. Sci.* **110**, 9824–9829 (2013).
53. Cael, B. B., Dutkiewicz, S. & Henson, S. A. Abrupt shifts in 21st-century plankton communities. *Sci. Adv.* **7**, 8593–8622 (2021).
54. Penuelas, J., Janssens, I. A., Ciais, P., Obersteiner, M. & Sardans, J. Anthropogenic global shifts in biospheric N and P concentrations and ratios and their impacts on biodiversity, ecosystem productivity, food security, and human health. *Glob. Chang. Biol.* **26**, 1962–1985 (2020).
55. Jiang, H.-B. *et al.* Ocean warming alleviates iron limitation of marine nitrogen fixation. *Nat. Clim. Chang.* **8**, 709–712 (2018).
56. Krishnamurthy, A., Moore, J. K., Mahowald, N., Luo, C. & Zender, C. S. Impacts of atmospheric nutrient inputs on marine biogeochemistry. *J. Geophys. Res.* **115**, G01006 (2010).
57. Talarmin, A. *et al.* Seasonal and long-term changes in elemental concentrations and ratios of marine particulate organic matter. *Global Biogeochem. Cycles* **30**, 1699–1711 (2016).
58. Tanioka, T., Fichot, C. G. & Matsumoto, K. Toward Determining the Spatio-Temporal Variability of Upper-Ocean Ecosystem Stoichiometry From Satellite Remote Sensing. *Front. Mar. Sci.* **7**, 604893 (2020).

59. Tanioka, T. *et al.* Global Ocean Particulate Organic Phosphorus, Carbon, Oxygen for Respiration, and Nitrogen (GO-POPCORN) data from Bio-GO-SHIP cruises. (2022) doi:https://doi.org/10.5061/dryad.05qfttf5h.
60. Garcia, C. A. *et al.* Linking regional shifts in microbial genome adaptation with surface ocean biogeochemistry. *Philos. Trans. R. Soc. B Biol. Sci.* **375**, 20190254 (2020).
61. Lee, J. A., Garcia, C. A., Larkin, A. A., Carter, B. R. & Martiny, A. C. Linking a Latitudinal Gradient in Ocean Hydrography and Elemental Stoichiometry in the Eastern Pacific Ocean. *Global Biogeochem. Cycles* **35**, (2021).
62. Lomas, M. W. *et al.* Sargasso Sea phosphorus biogeochemistry: An important role for dissolved organic phosphorus (DOP). *Biogeosciences* **7**, 695–710 (2010).
63. Becker, S. *et al.* GO-SHIP Repeat Hydrography Nutrient Manual: The Precise and Accurate Determination of Dissolved Inorganic Nutrients in Seawater, Using Continuous Flow Analysis Methods. *Front. Mar. Sci.* **7**, 908 (2020).
64. Baer, S. E. *et al.* Carbon and nitrogen productivity during spring in the oligotrophic Indian Ocean along the GO-SHIP IO9N transect. *Deep Sea Res. Part II Top. Stud. Oceanogr.* **161**, 81–91 (2019).
65. Lauvset, S. K. *et al.* A new global interior ocean mapped climatology: The 1° × 1° GLODAP version 2. *Earth Syst. Sci. Data* **8**, 325–340 (2016).
66. Key, R. M. *et al. Global ocean data analysis project, version 2 (GLODAPv2). Ornl/Cdiac-162, Ndp-093* (2015) doi:10.3334/CDIAC/OTG.NDP093_GLODAPv2.
67. Richardson, K. & Bendtsen, J. Vertical distribution of phytoplankton and primary production in relation to nutricline depth in the open ocean. *Mar. Ecol. Prog. Ser.* **620**, 33–46 (2019).
68. Brun, P. *et al.* Ecological niches of open ocean phytoplankton taxa. *Limnol. Oceanogr.* **60**, 1020–1038 (2015).
69. Holte, J., Talley, L. D., Gilson, J. & Roemmich, D. An Argo mixed layer climatology and database. *Geophys. Res. Lett.* **44**, 5618–5626 (2017).
70. R Core Team. R: A Language and Environment for Statistical Computing. (2021).
71. Wood, S. N. Fast stable restricted maximum likelihood and marginal likelihood estimation of semiparametric generalized linear models. *J. R. Stat. Soc. Ser. B Stat. Methodol.* **73**, 3–36 (2011).
72. Pedersen, E. J., Miller, D. L., Simpson, G. L. & Ross, N. Hierarchical generalized additive models in ecology: An introduction with mgcv. *PeerJ* **2019**, (2019).
73. Lindeman, R. H., Merenda, P. F. & Gold, R. Z. *Introduction to bivariate and multivariate analysis, Glenview, IL. Scott: Foresman and company* vol. 119 (1980).
