# Supplementary material for: Global patterns and predictors of C:N:P in marine ecosystems
Source: Commun Earth Environ. 2022 Nov 7;3(1):271. doi: 10.1038/s43247-022-00603-6 (PMC9640808; doi:10.1038/s43247-022-00603-6)
Supplement: Supplementary file 2 — Reporting Summary [file 43247_2022_603_MOESM2_ESM.pdf]

nature portfolio

Corresponding author(s): Adam Martiny, Tatsuro Tanioka

Last updated by author(s): Oct 6, 2022

Reporting Summary

Nature Portfolio wishes to improve the reproducibility of the work that we publish. This form provides structure for consistency and transparency in reporting. For further information on Nature Portfolio policies, see our Editorial Policies and the Editorial Policy Checklist.

Statistics

For all statistical analyses, confirm that the following items are present in the figure legend, table legend, main text, or Methods section.

n/a	Confirmed	
☐	☒	The exact sample size (*n*) for each experimental group/condition, given as a discrete number and unit of measurement
☐	☒	A statement on whether measurements were taken from distinct samples or whether the same sample was measured repeatedly
☐	☒	The statistical test(s) used AND whether they are one- or two-sided<br>*Only common tests should be described solely by name; describe more complex techniques in the Methods section.*
☐	☒	A description of all covariates tested
☐	☒	A description of any assumptions or corrections, such as tests of normality and adjustment for multiple comparisons
☐	☒	A full description of the statistical parameters including central tendency (e.g. means) or other basic estimates (e.g. regression coefficient) AND variation (e.g. standard deviation) or associated estimates of uncertainty (e.g. confidence intervals)
☐	☒	For null hypothesis testing, the test statistic (e.g. *F*, *t*, *r*) with confidence intervals, effect sizes, degrees of freedom and *P* value noted<br>*Give P values as exact values whenever suitable.*
☒	☐	For Bayesian analysis, information on the choice of priors and Markov chain Monte Carlo settings
☐	☒	For hierarchical and complex designs, identification of the appropriate level for tests and full reporting of outcomes
☒	☐	Estimates of effect sizes (e.g. Cohen's *d*, Pearson's *r*), indicating how they were calculated

Our web collection on statistics for biologists contains articles on many of the points above.

Software and code

Policy information about availability of computer code

Data collection	We use paired observations of particulate organic phosphorus (POP), nitrogen (PON), and carbon (POC) samples from 1970 stations collected between 2014 and 2020 as a part of a biological initiative for the Global Ocean Ship-Based Hydrographic Investigations Program (Bio-GO-SHIP)28,29. Samples used in this study are from cruises AMT-28, C13.5, I07N, I09N, NH1418, and P18 (Supplementary Table 1).
Data analysis	All processing and analysis was done in R version 4.1.0. Codes (data manipulation, analyses, figures, and tables) can be downloaded from the GitHub repository https://github.com/tanio003/CNPGlobal_paper_repo/tree/CommsEarthEnv.

For manuscripts utilizing custom algorithms or software that are central to the research but not yet described in published literature, software must be made available to editors and reviewers. We strongly encourage code deposition in a community repository (e.g. GitHub). See the Nature Portfolio guidelines for submitting code & software for further information.

Data

Policy information about availability of data

All manuscripts must include a data availability statement. This statement should provide the following information, where applicable:

- Accession codes, unique identifiers, or web links for publicly available datasets
- A description of any restrictions on data availability
- For clinical datasets or third party data, please ensure that the statement adheres to our policy

POM, hydrography, and metagenomes from Bio-GO-SHIP cruises used in this study are publicly available (ref. 28,59). Nutrient stress data of phytoplankton can be

accessed from the original publication cited in the main text (ref. 31). GLODAP version2.2016b data is publicly available (https://doi.org/10.5194/essd-8-297-2016). The model output from the CEMS2 Large Ensemble Simulation is available here (https://doi.org/10.26024/kgmp-c556).

Human research participants

Policy information about studies involving human research participants and Sex and Gender in Research.

Reporting on sex and gender	N/A
Population characteristics	N/A
Recruitment	N/A
Ethics oversight	N/A

Note that full information on the approval of the study protocol must also be provided in the manuscript.

Field-specific reporting

Please select the one below that is the best fit for your research. If you are not sure, read the appropriate sections before making your selection.

☐ Life sciences ☐ Behavioural & social sciences ☒ Ecological, evolutionary & environmental sciences

For a reference copy of the document with all sections, see nature.com/documents/nr-reporting-summary-flat.pdf

Ecological, evolutionary & environmental sciences study design

All studies must disclose on these points even when the disclosure is negative.

Study description	We collected and analyzed new POM samples across all major ocean basins as part of the biological initiative for the Global Ocean Ship-based Hydrographic Investigations Program or Bio-GO-SHIP (ref. 28,29).
Research sample	We collected 1970 paired POM samples (C, N, and P) in the top 30 m across a broad latitudinal range from 70°S to 50°N (Fig. 1, Supplementary Table 1) and analyzed them using consistent protocols.
Sampling strategy	Samples were collected across all major oceanic provinces from 70°S to 50°N using the consistent sampling method described previously (ref. 6,28,60,61). Briefly, 2-10 L seawater for the POM samples was collected from the onboard flow-through underway system at the sea surface (< 30 m) and was divided into POC/PON and POP triplicates after removing large plankton and particles using 30 µm nylon mesh. Each replicate was then filtered on precombusted Whatman GF/F filters with a nominal pore size of 0.7 µm. POP filters were rinsed with 5 mL of 0.17 M Na2SO4 prior to analysis to remove traces of dissolved organic phosphorus. All filtered POM samples were sealed in precombusted aluminum packets and were immediately frozen at -20 °C until analysis. The detection limit for POP measurement was ~ 0.3 µg.
Data collection	C.A.G, A.A.L, N.S.G, and A.J.F coordinated sample collection and/or processed samples. T.T, C.A.G, and A.A.L compiled metadata.
Timing and spatial scale	Samples used in this study are from cruises AMT-28, C13.5, I07N, I09N, NH1418, and P18 between 2014-2020 across 1970 stations. Samples were collected across all major oceanic provinces from 70°S to 50°N. Refer to Supplementary Table 1 for more details.
Data exclusions	Following the criteria used in Lee et al. (2021)61, we discarded any anomalous samples with POC:POP > 500, PON:POP < 1, and PON:POP > 100 after the stoichiometric ratios were calculated. These selection processes led to the 1970 final C-N-P paired POM measurements.
Reproducibility	Before POM sampling, all the carboys used were rinsed at least twice with the prefiltered underway seawater. The filtered volume of seawater was consistent between all POM (POC/N and POP) samples at each station and varied on a per station basis to ensure that the amount of collected material was minimally impacted by the difference in filtration time. Initial rinsing and the large sampling volume were aimed at reducing the effect of a time delay caused by the underway system.
Randomization	We binned data based on the latitude for statistical analyses used in Figures 3 and 4.
Blinding	This is not applicable in our study as the risk of bias that can be caused by an awareness of group assignment is minimal.

Did the study involve field work? ☐ Yes ☒ No

Reporting for specific materials, systems and methods

We require information from authors about some types of materials, experimental systems and methods used in many studies. Here, indicate whether each material, system or method listed is relevant to your study. If you are not sure if a list item applies to your research, read the appropriate section before selecting a response.

Materials & experimental systems

n/a	Involved in the study
☒	☐ Antibodies
☒	☐ Eukaryotic cell lines
☒	☐ Palaeontology and archaeology
☒	☐ Animals and other organisms
☒	☐ Clinical data
☒	☐ Dual use research of concern

Methods

n/a	Involved in the study
☒	☐ ChIP-seq
☒	☐ Flow cytometry
☒	☐ MRI-based neuroimaging
